# Preface

**DOI:** 10.1007/s11047-014-9480-3

**Published:** 2014-12-23

**Authors:** Dominik Ślęzak, Andrzej Skowron

**Affiliations:** Faculty of Mathematics, Informatics and Mechanics, University of Warsaw, ul. Banacha 2, 02-097 Warsaw, Poland

This special issue is dedicated to Information Granulation in Natural Computing. Natural Computing refers to computational processes taking place in nature and human-designed computing inspired by nature. When compound natural phenomena are analyzed in terms of computational processes, our understanding of the essence of computation is enhanced. On the other hand, Information Granulation is crucial for understanding compound, interactive and adaptive computations in nature. It enables one to pave the path from sensory measurements to concepts expressed in a natural-like language at a perception level responsible for understanding the analyzed situations or phenomena. Granular Computing provides a framework for modeling and reasoning about such concepts based on their approximations derived from the Information Granulation processes. The overall framework of Information Granulation includes already a number of real-world applications.

This special issue aims at emphasizing the role of Information Granulation in designing intelligent systems inspired by nature, performing computations through interactions with the physical objects in nature, and discovering characteristics of compound processes from both, large amounts of experimental data and interactively acquired information. The issue contains five papers selected out of eight submissions. The paper submissions were gathered on invitation basis and through the open call for papers. All submissions were carefully peer-reviewed by independent experts, with three evaluation reports per manuscript. One manuscript was rejected. Two paper submissions were withdrawn by authors due to the amount of time that would be required to address all reviewers’ remarks. The revised versions of five pre-accepted papers were reexamined by reviewers in order to assure that all necessary changes and extensions were thoroughly implemented.

The first paper, by Łukasz Maciura and Jan G. Bazan, is titled “Granular Computing in Mosaicing of Images from Capsule Endoscopy”. The authors discuss the algorithm which employs compound granules for construction of a mosaic from the images coming from an endoscope capsule. In order to apply the algorithm, combined images must have a common area where the correspondence of points is determined. This allows for determining the transformation parameters to compensate movement of the capsule that occurs between moments when the mosaic images were acquired. The developed algorithm for images from the capsule endoscopy has proved to be faster than commercial algorithms, while still comparably accurate. Ability to process data by means of granules gathering information related to compound medical concepts turns out to be crucial for the algorithm’s efficiency.

The second paper, by Bożena Kostek, Adam Kupryjanow, and Andrzej Czyżewski, is titled “Knowledge Representation of Motor Activity of Patients with Parkinson’s Disease”. The authors present an approach to the knowledge representation extraction from biomedical signals analysis concerning motor activity of Parkinson disease patients. This is done utilizing accelerometers attached to their bodies as well as exploiting video image of their hand movements. Experiments are carried out employing Artificial Neural Networks and Support Vector Machines for the recognition of characteristic motor activity disorders in patients. Obtained results indicate that it is possible to interpret some selected patient’s body movements with a sufficiently high effectiveness. Granular representation of signals turns out to be important for efficiency and robustness of this medical application as well.

The third paper, by Akinori Kanasugi, Akihiko Tsukahara, and Ki Ando, is titled “Hardware Implementation of Evolutionary Algorithms using Dynamic Reconfiguration Technology”. The authors utilize dynamic reconfiguration technology for the purpose of hardware implementation of evolutionary algorithms. Natural-computing-inspired algorithms are widely applied in data mining and knowledge discovery, including decision models based on information granulation as well. This paper considers two popular types of dynamic reconfigurations of genetic algorithms. The effectiveness of the proposed corresponding processor designed using VHDL is confirmed by thorough logical circuit simulations. Using the obtained framework for further acceleration of evolutionary-driven optimizations of information granulation models can be one of future steps in this area.

The fourth paper, by Romi Banerjee and Sankar K. Pal, is titled “Text Comprehension and the Computational Mind-Agencies”. The authors describe a computational mind framework for text comprehension in terms of Minsky’s theories of Society of Mind and Emotion Machine. The framework’s completeness is validated as a consequence of its representation of text understanding functions of the human brain, types of human memory and emulation of the layers of the mind. The hierarchies of information granules are present in most of derivations included in this paper. Besides considering their framework as a general text comprehender, the authors envision it as potentially useful in areas such as the design of intelligent plagiarism checkers, literature genre-cataloguers, differential diagnosis systems, and educational aids for children with reading disorders.

The fifth paper, by Hui Wang, Jiajin Huang, Erzhong Zhou, Zhisheng Huang, and Ning Zhong, is titled “Cognition-Inspired Route Evaluation Using Mobile Phone Data”. The authors propose a cognition-inspired route evaluation method to mine the intelligence of users in selecting a suitable route. It is based on observation that, usually, an experienced user knows which route is congested in a specified period of time and unblocked in another period of time. Moreover, a route used frequently and recently by a user is usually the suitable one to satisfy the user’s needs. The authors employ the ACT-R (Adaptive Control of Thought-Rational) method combined with the paradigms of processing granules of data, information and knowledge in order to model how users select suitable routes. Experiments show that such approach is effective and feasible to evaluate the suitability of the routes inspired by cognition.

In summary, we hope that this special issue illustrates how to utilize Information Granulation in practical applications related to modeling, classification and discovery, how to combine principles of Information Granulation and Nature-Inspired Computing, and how those principles can enhance each other.

We would like to express our appreciation to the authors of all submitted papers for their excellent contributions and to reviewers for their hard work and insightful remarks. We are also particularly grateful to Professor Grzegorz Rozenberg, the Founding Editor-in-Chief of the Natural Computing Journal, for his interest and great support for the idea of preparing this special issue.

